# Does Primary Care Mental Health Resourcing Affect the Use and Costs of Secondary Psychiatric Services?

**DOI:** 10.3390/ijerph110908743

**Published:** 2014-08-26

**Authors:** Minna Sadeniemi, Sami Pirkola, Maiju Pankakoski, Grigori Joffe, Raija Kontio, Maili Malin, Taina Ala-Nikkola, Kristian Wahlbeck

**Affiliations:** 1 Department of Psychiatry, Hospital District of Helsinki and Uusimaa, Porvoo Health Care Area, Porvoon psykiatrian poliklinikka, Kaivokatu 37, FI-06100 Porvoo, Finland; 2 Department of Mental Health and Substance Abuse Services, National Institute for Health and Welfare, Mannerheimintie 170, FI-00270 Helsinki, Finland; E-Mails: maiju.pankakoski@thl.fi (M.P.); maili.malin@thl.fi (M.M.); kristian.wahlbeck@thl.fi (K.W.); 3 Department of Psychiatry, University Hospital Region, Hospital District of Helsinki and Uusimaa, Välskärinkatu 12, PL 590, FI-00029 HUS, Finland; E-Mails: sami.pirkola@hus.fi (S.P.); grigori.joffe@hus.fi (G.J.); taina.ala-nikkola@hus.fi (T.A.-N.); 4 School of Health Sciences, University of Tampere, Medisiinarinkatu 3, FI-33014 Tampere, Finland; E-Mail: sami.pirkola@uta.fi; 5 Department of Psychiatry, Hospital District of Helsinki and Uusimaa, Hyvinkää Hospital Region, Vanha Valtatie 198, FI-04500 Kellokoski, Finland; E-Mail: raija.kontio@hus.fi

**Keywords:** costs and cost analysis, primary health care, nurses, psychiatric hospitals

## Abstract

Collaborative care models for treatment of depression and anxiety disorders in primary care have been shown to be effective. The aim of this study was to investigate at the municipal level to what extent investment in mental health personnel at primary care health centres in the study area is reflected in the costs and use of secondary psychiatric services. Furthermore, we analysed whether the service provision and use of secondary psychiatric care correlates with the socioeconomic indicators of need. We found significant variation in the amount of mental health personnel provided at the health centres, uncorrelated with the indicators of need nor with the costs of secondary psychiatric care. The amount of mental health nurses at the health centres correlated inversely with the number of secondary psychiatric outpatient visits, whereas its relation to inpatient days and admission was positive. The costs of secondary psychiatric care correlated with level of psychiatric morbidity and socioeconomic indicators of need. The results suggest that when aiming at equal access of care and cost-efficiency, the primary and secondary care should be organized and planned with integrative collaboration.

## 1. Introduction

Mental health services have been transformed from hospital-centred to a balanced model of integrated community-based services supported by psychiatric hospital care in most developed countries [1]. Since 1970, the number of psychiatric hospital beds in Finland has decreased by 80% [2]. The amount of inpatient days in psychiatric hospitals has decreased by 29% between 2002 and 2012. The number of psychiatric outpatient visits has increased between 2006 and 2012 by 30% [3]. Deinstitutionalization has not resulted in poorer outcomes [4], on the contrary, well developed community mental health services are associated with lower suicide rates than services oriented towards inpatient treatment provision [5,6].

In Finland, only approximately one-half of those suffering even from severe and comorbid mood disorders in the general population use health services for them [7]. For milder forms of disorders, the proportion of those receiving treatment may be even lower [8]. According to international studies, most of the patients with anxiety, mood and substance use disorders are diagnosed and treated in primary care [8,9]. In Finland, the role of primary care has been less prominent. In an earlier Finnish study, specialized mental health services accounted for two-thirds of the services that were used by people suffering from major depression and anxiety disorders [7].

In order to enhance the quality of care of psychiatric disorders in the primary care setting, effective models of collaborative care (CCM) for depression and anxiety have been developed [10,11,12,13]. These models typically include nurse care management and a greater degree of integration between primary and secondary care, and they are effective in improving patient outcome [13]. According to some studies, the CCM for treating depression is more effective than usual care but also results in greater treatment costs [14]. However, when indirect health care costs or productivity losses are also taken into account, the evidence seems to indicate, that CCM does not lead to a net increase in total health care costs [11,15].

In Finland, the municipalities provide primary health care at health centres, which form the point of system entry for people with mental health problems. Health centres provide prevention, diagnosis and treatment of common mental disorders, such as depression, anxiety disorders and alcohol use disorders. The health centres have a gatekeeper role, and access to specialized care is usually by reference from primary care only. For specialized public health services, including specialized mental health care, the municipalities form hospital districts.

During the last few years in Finland, mental health nurses have been increasingly included in primary health centre staff, mostly for treating depression [16,17]. This kind of collaborative care model has been recommended by the National Plan for Mental Health and Substance Abuse Work to be implemented in health centres nationwide [18]. The patients meet the nurse for evaluation, psychoeducation and support, and sometimes short-term psychotherapy like interpersonal psychotherapy. Sometimes collaboration with a psychiatrist is also included in the care model. According to the Finnish Current Care Guideline for Treatment of Depression [19], complicated or treatment-resistant moderate episodes and severe depressive episodes should be treated in secondary psychiatric care. The degree to which health centres in Finland have special mental health care staff, as mental health nurses and psychologists, has not been studied prior to this study. Low socioeconomic status is generally associated with higher psychiatric morbidity [20,21,22]. It is not known whether this is taken into account when organizing primary care psychosocial services in Finland.

This study is a part of the European REsearch on FINancing systems’ Effect on the quality of MENTal health care (REFINEMENT) project [23]. The aim of this study was to: (1) investigate the association of the amount of mental health personnel provided at health centres with the use and costs of secondary psychiatric services in the study area and (2) investigate whether the investment in psychosocial resources at primary care level, and the use and costs of secondary psychiatric care reflect the needs of the population as measured by socioeconomic indicators. The first hypothesis: (1) was that the more resources there are for mental health care in the primary care health centres, the less need there is for psychiatric services at the secondary level, as measured by psychiatric hospital admissions, inpatient days and outpatient visits, and there will be less costs for the municipality for secondary psychiatric care. We also hypothesized: (2) that certain indicators of social disadvantage and psychiatric morbidity would associate with higher service resourcing at the primary care level, and higher use of secondary psychiatric care.

## 2. Experimental Section

In the first phase of the REFINEMENT project mental health services were classified in nine European countries, one study area in each country, by using the European Service Mapping Schedule—Revised (ESMS-R) service mapping tool [24,25,26,27,28]. In Finland, addiction services were also mapped. The mapping was restricted to adult services. The mapping was done at the beginning of 2012.

The study area is one of the hospital districts in Finland, *i.e.*, the Hospital District of Helsinki and Uusimaa (HUS). It is owned and governed by 26 municipalities in the southernmost part of Finland, the Uusimaa district. The total population in the HUS area is 1.5 million people (adult population (18+) is 1.2 million), which is over a quarter of the entire population of Finland. The adult population in individual municipalities varies from 1200 to 490,000. There are 18 municipal health centres in the HUS area. Smaller municipalities have joint health centres. The health centre in Helsinki also offers inpatient psychiatric care. With some exceptions, the other municipalities purchase inpatient services from the HUS hospital district.

The amount of primary care psychiatric personnel was expressed as the number of nurses, psychologists, other mental health workers and psychiatrists located at the health centres per 10,000 adult inhabitants of the municipality. Although we looked at the total amount of personnel, the mental health nurses were the most relevant and prevalent occupational group.

To define the use of secondary psychiatric care in 2012, for each municipality the number of psychiatric outpatient visits, the number of hospital admissions, the average length of the hospital stay and the total number of inpatient days were collected from Ecomed, the HUS hospital district register. The municipality of Hyvinkää provides secondary psychiatric outpatient care as a part of its own function, and the capital city of Helsinki has a major psychiatric unit providing both outpatient and inpatient secondary level psychiatric care. Both of these municipalities also buy psychiatric services from HUS. The care given at their own psychiatric units were added to the HUS statistics.

The total costs of the psychiatric secondary care provided by HUS in 2012 for each municipality were collected from the HUS financial management database, HUS-Total. The costs were expressed as euros per adult inhabitant in the year 2012. The costs of psychiatric care in the Hyvinkää and Helsinki municipal secondary psychiatric units were asked separately from their administration, and then added to the costs of psychiatric care provided by HUS for these municipalities. Thus, for secondary psychiatric services, the results reflect the psychiatric services provided by the Hospital District HUS and the services provided by the Hyvinkää and Helsinki municipal secondary psychiatric units.

Socioeconomic indicators of need for psychiatric services, as the unemployment rate, the average income per adult, the level of education and the percentage of single households were collected from the Finnish Statistics and Indicator Bank (SOTKA-net) [29] using data from 2011.

As a likely indicator of psychiatric morbidity, a crude mental health index (MHI) for each municipality is provided by the National Institute for Health and Welfare. The index is based on the incidence of suicides and suicide attempts, the number of persons eligible for special reimbursement for antipsychotic medication, and the number of persons on disability pension due to mental disorders in a given area [30]. Each of the indicators is given equal weight. The MHI is counted as an average over three years. The MHI represents an effort to utilize national statistics, and the National Institute of Health and Welfare promotes its’ use in national policy making and resource allocation. However, the MHI has not previously been studied. Similar but not identical measures have been presented and studied in other countries [31].

Because of the small number of municipalities, Spearman correlations were used to investigate the association between the indicators of need, the MHI, the amount of psychiatric personnel, the number of nurses at primary care level and the use and costs of secondary psychiatric care. Linear regression was used in assessing the joint effect of more than one variable (MHI, number of primary care mental health nurses, secondary care psychiatric outpatient visits) on psychiatric inpatient days and admission rate. Analyses were performed using SPSS version 21 (IBM Corp., Armonk, NY, USA).

## 3. Results and Discussion

### 3.1. Results 

**Figure 1:**
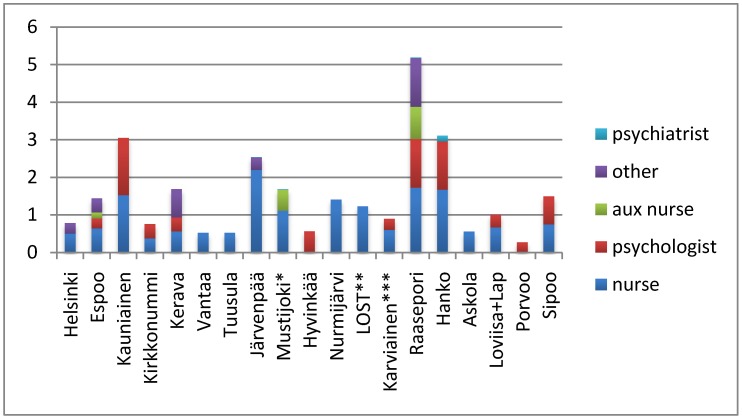
The number of mental health nurses, psychologists, auxiliary nurses (aux nurse), other mental health workers (other) and psychiatrists in the primary care health centres of municipalities per 10,000 adult inhabitants.

Notes: Joint municipal authorities covering the following municipalities: * Mäntsälä, Pornainen, ** Lohja, Siuntio, Inkoo, Karjalohja, *** Karkkila, Nummi-Pusula, Vihti.

There was significant variation between the municipalities in the amount of mental health personnel in the health centres (Figure 1). The smallest amount of personnel was observed in the Porvoo health centre, with only one psychologist for the entire adult population of approximately 38,000. The highest amount of mental health personnel was in Raasepori primary health care.

**Figure 2:**
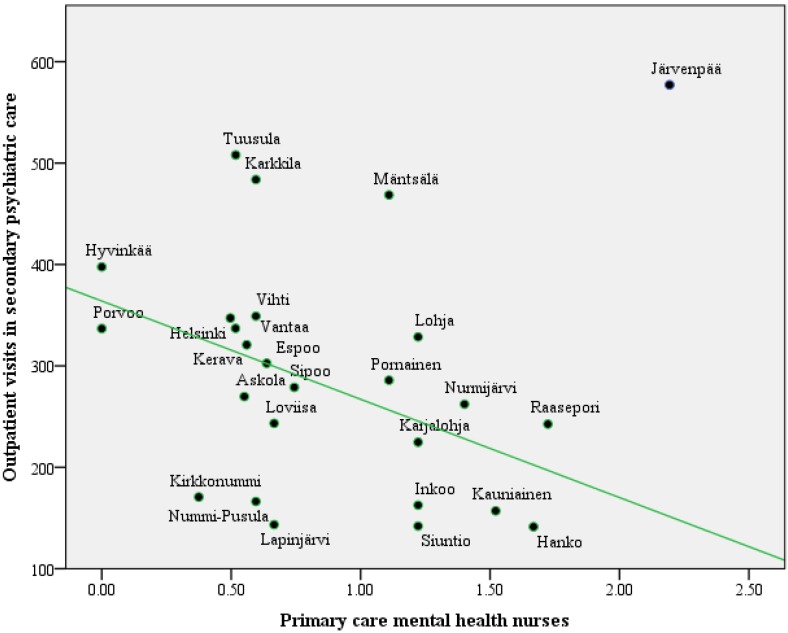
Primary care mental health nurses and outpatient visits in secondary psychiatric care. Each dot represents a municipality. Järvenpää was excluded from the calculation of the regression line.

In the analysis of the study variables (Table 1), the number of mental health nurses in primary care correlated negatively with the number of outpatient visits in secondary psychiatric care (Figure 2). There is a significant negative correlation (Spearman correlation coefficient −0.39, *p* = 0.050) between the number of primary care mental health nurses per 10,000 adults and the number of outpatient visits per 1000 adults per year in secondary psychiatric care. The municipality of Järvenpää is an outlier, having both heavy staffing of nurses in primary care and a great number of outpatient visits in secondary psychiatric care. If it is left out of the analysis, the correlation coefficient is larger (−0.56, *p* = 0.003). The total amount of mental health personnel did not show any correlation with the other variables.

**Table 1:** Correlations between the variables.

Variable	Primary Care Mental Health Nurses	Outpatient Visits	Inpatient Days	Admission Rate	Length of Stay	Costs	MHI	Unemployment Rate	Average Income	Single Households
Primary care mental health nurses	1									
Outpatient visits in secondary psychiatric care	−0.39 *	1								
Psychiatric inpatient days	0.19	0.41 *	1							
Psychiatric hospital admissions	0.074	0.36	0.68 **	1						
Length of hospital stay	0.07	−0.007	0.21	−0.55 **	1					
Costs of secondary psychiatric care	−0.075	0.63 **	0.83 **	0.73 **	−0.041	1				
MHI	−0.22	0.29	0.39	0.62 **	−0.39 *	0.69 **	1			
Unemployment rate	−0.19	0.06	0.37	0.54 **	−0.31	0.46 *	0.64 **	1		
Average income	−0.079	0.17	−0.065	−0.43 *	0.57 **	−0.18	−0.45 *	−0.66 **	1	
Percentage of single households	−0.056	0.11	0.47 *	0.51 **	−0.16	0.61 **	0.75 **	0.82 **	−0.49 *	1
Education	−0.096	0.17	−0.088	−0.44 *	0.56 **	−0.13	−0.37	−0.66 **	0.95 **	−0.41

Notes: * Correlation is significant at the 0.05 level; ** Correlation is significant at the 0.01 level.

A significant positive correlation of the number of secondary psychiatric care outpatient visits with the number of psychiatric inpatient days was observed. Multivariate analysis was used to control for confounding factors in investigating associations with hospitalizations. A linear regression model adjusted with secondary psychiatric outpatient visits and MHI shows a positive association between number of primary care mental health nurses and inpatient days (Table 2). Adjusted with the same variables, a positive association between number of primary care mental health nurses and hospital admissions was seen. In the bivariate analyses, these associations between the number of nurses and inpatient days and hospital admissions were not seen.

**Table 2:** Regression models explaining inpatient days and admission rate by MHI, number of primary care psychiatric nurses and secondary care psychiatric outpatient visits.

Independent Variables	Inpatient Days			Admission Rate		
	B	se	*p*	B	se	*p*
Intercept	−23.27	53.23	0.666	−5.06	3.13	0.12
MHI	1.23	0.67	0.077	0.15	0.04	0.001
Primary care mental health nurses	44.23	17.94	0.022	2.29	1.06	0.041
Outpatient visits in secondary psychiatric care	0.16	0.08	0.074	0.00	0.01	0.485

**Figure 3:**
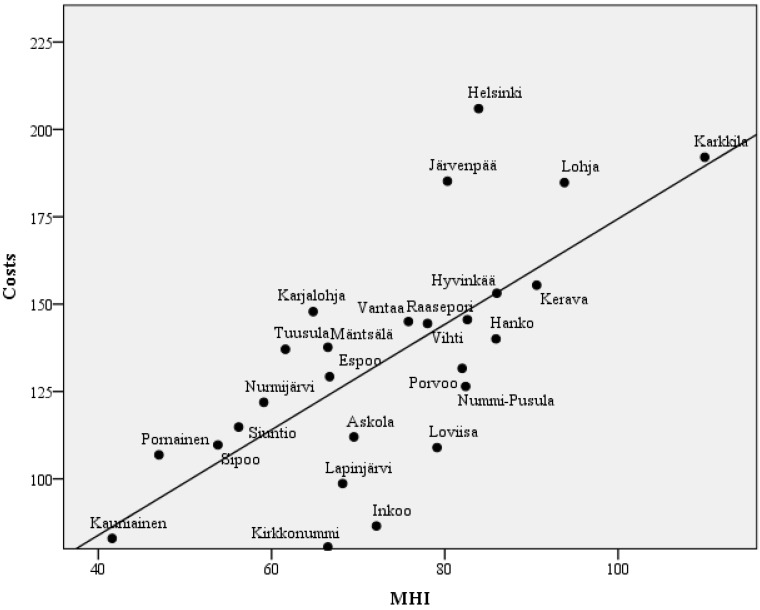
The costs of secondary psychiatric care in relation to the Mental Health Index (MHI). Each dot represents a municipality. Costs are expressed as euros per adult inhabitant. The correlation is significant (c = 0.69, *p* < 0,001).

The number of mental health nurses in primary care did not correlate with the total costs of secondary psychiatric care nor with the socioeconomic indicators or the MHI. The total costs of secondary psychiatric care correlated significantly with the MHI (Figure 3), as well as with the unemployment rate, the percentage of single households, and expectedly the number of psychiatric admissions, inpatient days and outpatient visits. The admission rate significantly correlated positively with the unemployment rate, the proportion of single households and the MHI. A significant negative correlation was found between the admission rate and the average income, the level of education and the average length of the hospital stay. No reciprocal relationship with outpatient visits and inpatient days, admission rates or length of hospital stay was found.

### 3.2. Discussion 

In this study in investigating the mental health service system in an urban, semi-urban region in Finland, we found significant variation in processes and costs of organizing services. We found that a higher provision of mental health nurses, but not the total amount of mental health personnel, at primary care level associates with decreased use of specialized psychiatric outpatient visits. Interestingly, higher number of primary care psychiatric nurses at health centres associates with increased use of psychiatric inpatient care. This may partly explain why, contradictory to our hypothesis, higher primary care mental health resources did not associate with lower total costs of secondary psychiatric care. In addition, our analyses are suggesting that the Mental Health Index, derived from statistics data on suicides, psychiatric medication reimbursements and disability pension due to mental disorders, may be a valid and feasible indicator for cost drivers of secondary psychiatric care.

Previous studies on collaborative care have mostly been clinical studies. Our system-level study shows that models of using mental health personnel in primary care have been quite widely implemented in everyday clinical practice in the study area, despite the lack of an overall integrated policy strategy. Wide, more than tenfold, variation in per capita primary care psychosocial resources was found in the study area. Municipalities have independently developed service provision patterns and systems of their own. The differences in the amount of staffing does not seem to derive from need for services, at least not as measured by the mental health index or the socioeconomic indicators.

Primary care psychiatric resourcing does not seem to relate to the total costs of specialized psychiatric care in the study area. Rather, the costs of specialized psychiatric care associate with the MHI, the unemployment rate and the percentage of single households. According to this study, mental health nurses are the most prevalent and relevant occupational group in primary care. Increasing their number decreases the use of secondary psychiatric outpatient care. However, it seems that investment in provision of mental health services in primary care is linked to an increase of psychiatric inpatient care, which is the main cost driver in mental health care provision. Perhaps, without integration with secondary level psychiatric services, the primary level mental health nurses have a potential to recognize needs that in a more integrated context could be met also in outpatient setting. It may also be that mental health services in primary care cater to a different clinical patient population than inpatient psychiatric services. Our study indicates a need to further develop mental health services in primary care to the most vulnerable groups, as simply adding resources to primary care seems to be an inefficient way to reduce psychiatric inpatient care needs.

The positive independent association of higher number of primary care mental health nurses and psychiatric hospital admissions and inpatient days is interesting and somewhat surprising. It may indicate that the primary care level, in contrast to secondary level of psychiatric outpatient services, lacks the capacity to respond to more complex and advanced needs of patients. In some cases this may lead to increased hospital use. Thus, quality and content of care may be an issue in primary care settings, especially in small health centres. A small unit cannot provide specialized services. Another explanation would be that more primary care psychiatric nurses means increased recognition of unmet needs for inpatient care.

To ensure horizontal equity in access to mental health care, evaluation of mental health policy should be based on concurrent evidence-based assessment of the organization and use of services in relation to indicators of social deprivation [32]. The psychiatric hospital admission rates have, in studies from Northern Europe, been found to differ considerably according to the level of socioeconomic deprivation [33]. In some studies, the length of stay has been found to correlate negatively with socioeconomic deprivation [34]. According to our study, the costs of psychiatric secondary care correlate clearly with MHI, which seems understandable and may be interpreted as a necessity. In the municipalities with a high MHI and high unemployment, there are more admissions and the hospital stays are shorter, which is in line with previous findings. In the more prosperous municipalities with a lower MHI, there are less admissions but the stays are longer. This may point to a level of inequality in inpatient care, which might merit further investigation. In some cases, the availability of resources may also to some extent determine service utilization. As to primary care mental health resources, it is worth asking whether they should better reflect the socioeconomic indicators of need and be organized in larger units rather than small municipalities. To assure cost-efficiency, primary care and secondary psychiatric care should be developed as a whole. This would ensure citizens equal access to both low threshold care and specialized psychiatric care.

### 3.3. Strengths and Limitations 

The strength of this study is that it is the first study where the primary care mental health service system has been systematically mapped in a relatively large and diverse area in terms of service settings. It is also the first study where the costs and use of secondary psychiatric care has been studied in relation to the MHI and socioeconomic indicators.

Since this study was a cross-sectional study of a given mental health care system and no outcome measures or quality of care measures were used, it is not possible to compare the actual cost-effectiveness of care. Also, no analysis of the type of care actually provided by the primary care psychiatric nurses was included in this study. So, the degree to which the care meets the criteria for CCM remains unknown.

With this complex, cross-sectional and naturalistic study setting we are unable to make direct causal conclusions, but rather present significant and possibly important associations. However, need for research data for the use of service development is emerging and our findings implicate further outcome studies.

## 4. Conclusions 

Providing primary care mental health nurses, in an attempt for collaborative care, associates with decreased use of psychiatric outpatient care and increased psychiatric hospital use. Regional socioeconomic disadvantage or psychiatric morbidity associate with higher costs, but does not seem to relate to planning of collaborative care models. On the basis of complex and non-convergent effects, a more integrated management of service provision model seems justified.
